# Supplementary material for: Effect of Supplementing Hydrolysable Tannins to a Grower–Finisher Diet Containing Divergent PUFA Levels on Growth Performance, Boar Taint Levels in Back Fat and Intestinal Microbiota of Entire Males
Source: Animals (Basel). 2019 Dec 2;9(12):1063. doi: 10.3390/ani9121063 (PMC6940899; doi:10.3390/ani9121063)
Supplement: Supplementary file 1 [file animals-09-01063-s001.pdf]

Table S1. Sequences and accession numbers of primers used for the quantitative Real-Time PCR.

Target genes[1]	Sequences (Forward/Reverse)	Accession Number	Ta (°C)	Product length (bp)
Cyp1A1	GCAGCCACCTTGGAATCTCT / AGGTCCTGACCACCCAGAAT	NM_214412.1	65.3	126
Cyp1A2	TCCTGAGGAAAATGGTCCAG / GTATCAAATCCGGCTCCAAA	NM_001159614.1	61.1	175
Cyp2A19	GCCACTTTCGACTGGCTCT / CTCCTCGATACCCCGCTTG	NM_214417.1	64.8	129
Cyp2E1	CTGGAGGCACTCAGGAAGAC / CTTCCAGGCAGGTAGCGTAG	NM_214421.1	64.9	230
Cyp3A29	ATACGGGCACTAGTGGAAGC / TACTAGGTGGGGGTGGATGG	NM_214423.1	64.7	83
GAPDH	GTCGGTTGTGGATCTGACCT / AGCTTGACGAAGTGGTCGTT	NM_001206359.1	64.6	210

[1]Cyp = Cytochrome; GADPH = Glyceraldehyde 3-phosphate dehydrogenase, used as housekeeping gene; Ta = Annealing temperature ; bp = base pairs

Table S2. Effect of dietary hydrolysable tannin and PUFA level on feeding behavior of grower-finisher pigs[1].

	**H**	**L**	**H**	**L**		**P-values[2]**		
**Item**	**-**	**-**	**+**	**+**	**SEM**	**T**	**P**	**T × P**
Visits of the feeder, n								
Total	704	922	591	821	59.8	0.09	< 0.01	0.92
Daily	7.3	9.5	6.0	8.4	0.64	0.08	< 0.01	0.91
Time at the feeder, min								
Total	5283	5537	5396	5391	174.1	0.91	0.42	0.40
Daily	54.3	56.9	55.3	55.2	1.95	0.82	0.43	0.38
Per visit	7.8	6.4	9.5	6.7	0.46	0.04	< 0.01	0.16
Feed intake, g								
Per visit	314	232	358	256	21.7	0.11	< 0.01	0.64
Per min	39	36	38	38	1.8	0.84	0.20	0.06

[1] H = high dietary PUFA level; L = low dietary PUFA level; - = without hydrolysable tannin supplementation; + = with hydrolysable tannin (3%) supplementation.

[2] Probability values for hydrolysable tannin supplementation (T), dietary PUFA level (P) and T × P interaction.

Table S3. Effect of dietary hydrolysable tannin supplementation and PUFA level on carcass characteristics determined via dissection and DXA and organ weights of grower-finisher pigs[1].

Item	H	L	H	L	SEM	P-values[2]		
	-	-	+	+		T	P	T × P
Hot carcass weight, kg								
Carcass yield, %	79.71	80.33	80.06	80.54	0.305	0.32	0.05	0.80
Cold Loss,%[3]	1.92	1.72	2.24	1.66	0.251	0.59	0.12	0.44
Lean meat, %[4]	58.13	58.87	58.14	56.93	0.707	0.45	0.23	0.44
Loin	27.01	26.70	26.86	26.89	0.265	0.91	0.52	0.44
Ham	18.40	18.54	18.33	17.70	0.318	0.16	0.44	0.23
Shoulder	12.75	12.69	12.92	12.31	0.287	0.69	0.19	0.28
Belly	16.56	16.62	16.41	16.23	0.249	0.19	0.75	0.55
Backfat, %	7.35	7.09	7.14	7.42	0.374	0.84	0.98	0.43
10th rib backfat thickness, mm	20.49	17.04	17.65	18.01	1.346	0.40	0.17	0.09
Subcutaneous fat, %[5]	12.68	12.46	12.35	12.68	0.503	0.91	0.90	0.54
Omental fat, %[6]	1.04	1.02	1.02	1.13	0.084	0.54	0.60	0.38
DXA measurements								
Total mass, kg	42.29	41.30	40.68	41.73	1.514	0.63	0.98	0.40
Bone mass, kg	1.09	1.05	1.05	1.08	0.035	0.74	0.96	0.52
Fat mass, kg	7.48	7.09	7.16	7.56	0.578	0.88	1.00	0.45
Lean mass, kg	33.70	33.16	33.47	33.12	1.060	0.44	0.95	0.47
Organ weight, g								
Liver	1663	1643	1482	1506	0.051	< 0.001	0.95	0.60
Kidney	320	299	310	306	0.011	0.88	0.24	0.39
Testis	522	538	490	472	0.040	0.14	0.97	0.62
Bulbourethral gland	153	149	134	138	0.013	0.16	0.98	0.67
Salivary gland	69	75	71	72	0.005	0.87	0.39	0.53

1 H = high dietary PUFA level; L = low dietary PUFA level; - = without hydrolysable tannin supplementation; + = with hydrolysable tannin (3%) supplementation; DXA = Dual-Energy X-ray Absorptiometry measurements performed in human thick mode. 2 Probability values for hydrolysable tannin supplementation (T), dietary PUFA level (P) and T × P interaction. 3 Weight loss of the hot carcass during chilling at 2°C for 24 h. 4 Sum of denuded shoulder, loin, and ham weight as a percentage of cold carcass weight. 5 Sum of external fat from the shoulder, loin, and ham expressed as a percentage of cold carcass weight. 6 Omental fat weight expressed as a percentage of cold carcass weight.

Table S4. Effect of dietary hydrolysable tannin and PUFA level on meat quality traits of the loin and androstenone, skatole and indole level in the loin and adipose tissue of grower-finisher pigs[1].

	**H**	**L**	**H**	**L**		**P-values[2]**		
**Item**	**-**	**-**	**+**	**+**	**SEM**	**T**	**P**	**T × P**
pH								
45 min	6.50	6.18	6.50	6.24	0.208	0.91	0.17	0.90
24 h	5.54	5.56	5.56	5.50	0.028	0.42	0.37	0.13
Temperature								
45 min	6.50	6.18	6.50	6.24	0.208	0.91	0.17	0.90
24 h	4.75	4.88	4.39	4.95	0.356	0.58	0.19	0.41
Color[3]								
L*	47.17	46.67	47.64	49.21	0.755	0.04	0.45	0.15
a*	5.36	5.09	4.78	4.81	0.287	0.08	0.62	0.55
b*	2.58	2.35	2.55	2.76	0.250	0.29	0.96	0.23
Chroma value	5.96	5.63	5.45	5.57	0.348	0.30	0.71	0.41
Water-holding capacity, %								
Drip loss	2.01	2.18	2.06	2.38	0.161	0.36	0.10	0.60
Thaw loss	5.70	6.81	5.85	5.52	0.755	0.46	0.61	0.35
Cook loss	24.73	24.79	25.67	25.32	0.549	0.09	0.73	0.63
Total loss	31.80	32.67	33.65	33.30	0.687	0.04	0.65	0.30
Shear force, kg	8.00^{xy}	8.17^{xy}	8.85^{y}	7.41^{x}	0.432	0.91	0.15	0.07

^{x,y} Values within a row with different superscripts tend to differ significantly at $P \leq 0.10$.

1 H = high dietary PUFA level by including 2% soy oil; L = low dietary PUFA supplementation by including 2% tallow; - = without hydrolysable tannin supplementation; + = with hydrolysable tannin (3%) supplementation

2 Probability values for hydrolysable tannin supplementation (T), dietary PUFA level (P) and T × P interaction

3 L* = lightness (greater values equal lighter color); r* = Redness (greater values equal redder color); b* = yellowness (greater values equal more yellow color); chroma value (color saturation) = $\sqrt{a*^{2} + b^{*2}}$

Table S5. DNA purity and quality per each sample.

Animal	DNA purity	GQN[1]
**8755**	1.9	3.5
**8756**	1.96	4.6
**8758**	2.07	3.1
**8759**	1.88	4.1
**8768**	2	1.9
**8769**	2	2.8
**8771**	1.89	4
**8772**	1.96	5.1
**8781**	1.96	3
**8783**	1.95	3.7
**8786**	2.05	5.2
**8794**	1.85	4.3
**8797**	1.85	3.7
**8799**	2.01	3.8
**8805**	1.93	3.6
**8806**	2.03	4
**8807**	1.92	4.1
**8808**	2.02	3.5
**8812**	1.96	4.4
**8813**	2.05	2.3
**8815**	1.79	4.7
**8820**	1.85	5.1
**8822**	1.99	5.2
**8823**	2.04	4.8
**8826**	2.13	3.2
**8831**	1.83	2.9
**8832**	2.03	4.2
**8835**	2.2	2.9
**8836**	1.9	3.1
**8847**	1.91	2.4
**8848**	2.09	2.9
**8849**	2.14	3.3
**8852**	2.13	5.1
**8853**	1.86	5.3
**8854**	2.05	4.9
**8855**	2.14	5.8
**8860**	2.07	4.9
**8861**	2	6.3
**8862**	1.82	4.7
**8863**	1.89	4.7
**8886**	2.07	3.5
**8888**	1.94	4.9
**8889**	1.97	5.1
**8890**	1.97	3.5

[1]GQN = Genomic Quality Number, to assess the quality of gDNA
